# Topological edge and corner states in coupled wave lattices in nonlinear polariton condensates

**DOI:** 10.1515/nanoph-2023-0556

**Published:** 2024-02-02

**Authors:** Tobias Schneider, Wenlong Gao, Thomas Zentgraf, Stefan Schumacher, Xuekai Ma

**Affiliations:** Department of Physics and Center for Optoelectronics and Photonics Paderborn (CeOPP), Paderborn University, 33098 Paderborn, Germany; Eastern Institute for Advanced Study, Eastern Institute of Technology , Ningbo, Zhejiang , 315200, China; Institute for Photonic Quantum Systems (PhoQS), Paderborn University, 33098 Paderborn, Germany; Wyant College of Optical Sciences, University of Arizona, Tucson, AZ 85721, USA

**Keywords:** topological edge states, topological corner states, exciton polaritons, optical multistability, AAH chains, higher-order topological insulators

## Abstract

Topological states have been widely investigated in different types of systems and lattices. In the present work, we report on topological edge states in double-wave (DW) chains, which can be described by a generalized Aubry-André-Harper (AAH) model. For the specific system of a driven-dissipative exciton polariton system we show that in such potential chains, different types of edge states can form. For resonant optical excitation, we further find that the optical nonlinearity leads to a multistability of different edge states. This includes topologically protected edge states evolved directly from individual linear eigenstates as well as additional edge states that originate from nonlinearity-induced localization of bulk states. Extending the system into two dimensions (2D) by stacking horizontal DW chains in the vertical direction, we also create 2D multi-wave lattices. In such 2D lattices multiple Su–Schrieffer–Heeger (SSH) chains appear along the vertical direction. The combination of DW chains in the horizonal and SSH chains in the vertical direction then results in the formation of higher-order topological insulator corner states. Multistable corner states emerge in the nonlinear regime.

## 1 Introduction

Recent years have seen a surge on the study of phases of matter classified as topological in various physical platforms [1], [2], [3], [4]. Despite being originally conceived in condensed matter systems for electrons, topological states have been reported in various photonic systems such as photonic crystals, coupled waveguides, metamaterials, optomechanics, and exciton-polaritons [5]. Such topological states have shown prospect for bringing the coveted topologically protected robustness into various applications, for instance unidirectional signal propagation [6], [7], optical communication, single mode lasing [8], vertical-cavity surface-emitting lasers (VCSELs) [9], quantum information processing [10], [11], just to name a few. Morover, photonic techniques were used to further extend the realm of topological states, some of which are not readily accessible in condensed matter systems, for instance Floquet topological insulators [12], synthetic space topological insulators [13], [14], Thouless pumping [15], and nonlinear topological insulators [16].

Besides the study of novel types of topological structures, one aspect that deserves further attention is the interplay between topology and nonlinearity. One platform of significant current interest in which both topology and nonlinearity can be conveniently investigated and controlled are so-called exciton polaritons. Exciton polaritons are partial-light partial-matter quasi-particles, originating from the strong coupling of excitons and photons in (planar) microresonators [17]. The hybrid nature gives rise to a strong polariton-polariton interaction, which is one of the main factors for the realization of polariton condensation [18], [19], nonlinear functional polaritonic elements [20], [21], [22], [23], and enables the nonlinear control and trapping of macroscopically coherent polariton states in optically induced potential landscapes [24], [25], [26], [27]. In tailored lattice structures, different topological edge states can be realized in the nonequilibrium polariton system such as in honeycomb lattices [28], [29], [30], Lieb lattices [31], [32], kagome lattices [33], [34], and Su–Schrieffer–Heeger (SSH) chains [8], [35], [36]. It was also reported that the strong polariton nonlinearity can induce novel topological phenomena such as multistable vortices [22], [37], topologically protected solitons [33], [38], gap solitons [39], coupling between corner modes in higher-order polariton insulators [40], bistable topological insulators [41], [42], and the double-sided skin effect [43].

One prominent finding in quasi one-dimensional (1D) topological systems is the characteristic zero-dimensional (0D) localization of states on the edges. To systematically obtain such topological states in 1D systems, one possible route is by referring to the periodic table that classifies the ten discrete symmetry classes that coincide with the Altland-Zirnbauer (AZ) classification of random matrices [44]. For instance the topological group of the BDI class (one of the symmetry classes in the AZ table) is Z , corresponding to a winding number in the first homotopy group. Such states, however, often find obstacles in their realization in photonic systems, due to their stringent symmetry requirements. A more feasible realization is the renowned SSH model, in which the introduction of both chiral and geometrical mirror symmetry endows a finer topological class beyond the AZ table. With its generalizations, the SSH model has been extensively studied in various photonic systems [8], [45], [46], [47], [48]. It has been reported that a two-dimensional (2D) SSH model supports higher-order topological insulator states [49], i.e., corner states, which recently triggered broad interest [50], [51], [52], [53]. A less intensely studied but not less promising route is the Aubry-André-Harper (AAH) model [54] which translates into a Chern class in synthetic space [55]. Such kind of model has recently been proposed in polariton systems in 1D by using optically induced potentials [56].

**Figure 1:**
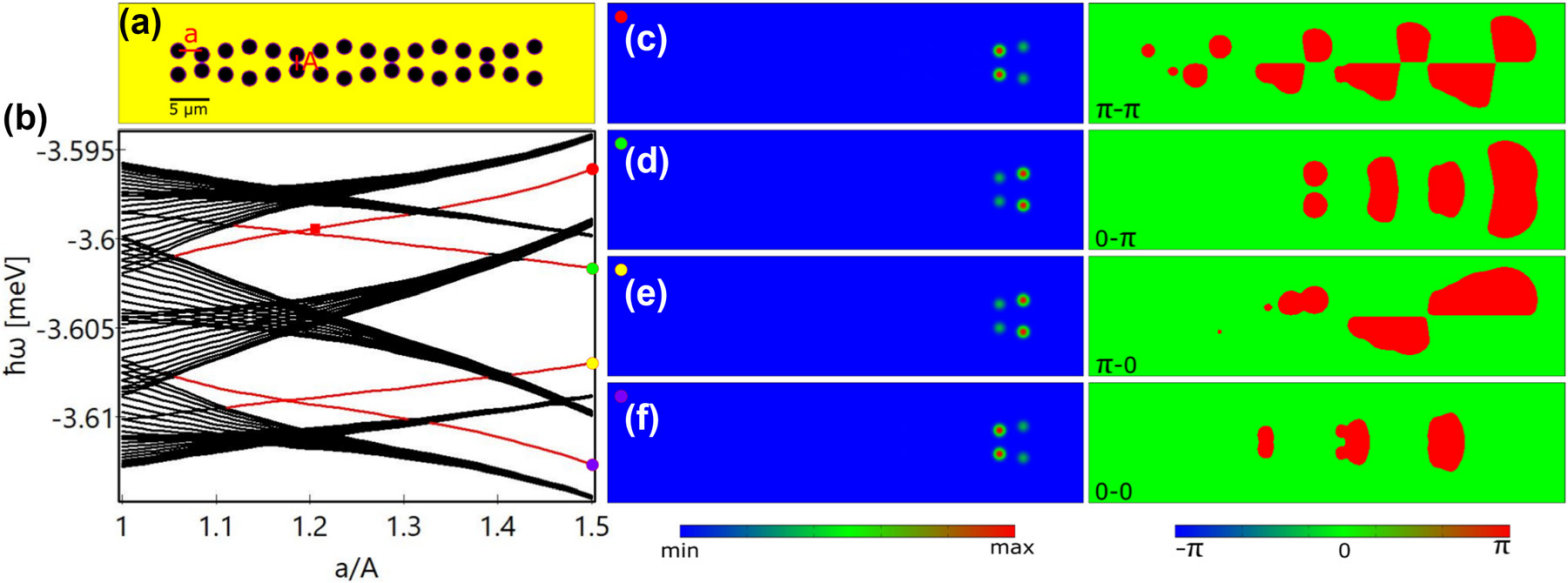
Edge states in double-wave (DW) chains. (a) DW potential chain with *a* = 3 μm, *A* = 2 μm, and wave modulation amplitude *d* = 0.5 μm. (b) Dependence of the energies (relative to the polariton dispersion minimum) of the linear eigenstates in the DW chain on the inter-wave separation *A* with *a* = 3 μm and *d* = 0.5 μm fixed. It is calculated based on a longer DW chain that contains 52 potential wells in each wave, instead of the 16 shown in (a), to make the energy bands and band gaps easier to distinguish. Red lines indicate the topological edge states and black lines are the bulk states. (c–f) Amplitude (left) and phase (right) distributions of the edge states marked in (b).

In the present work, we introduce and investigate a specific class of lattices to realize topological edge states: double-wave (DW) chains as sketched in Figure 1(a), which can be described in the framework of a coupled AAH model, i.e., cosinusoidal modulated potentials. First we discuss the topology of these structures based on a general tight-binding model Hamiltonian. Then we proceed to a specific physical realization in a planar semiconductor microcavity system with polaritonic excitations. We find that the edge states in the proposed DW chains become clearly distinguishable from the bulk states when the intra-wave separation between the potential wells is larger than the separation of the two waves, i.e., the inter-wave separation. By changing the specific structure of the waves, different edge states can be obtained, and they can even appear at different edges. Besides these edge states intrinsic to the structure, we find that the nonlinearity can transform specific bulk states into states that are localized at the edges. Moreover, in the nonlinear regime, the eigenstates on the edge with different phase distributions can be excited simultaneously, resulting in a final state where asymmetric edge states are stabilized by the nonlinearity. Several of these edge states can be stabilized for the same optical pumping parameters, resulting in a multistability that offers potential applications, e.g., in all-optical switching. Regularly stacking multiple identical DW chains into a 2D structure results in multiple SSH chains in the direction perpendicular to the DW chains. There, we demonstrate that the topology of the nontrivial DW chain and the nontrivial SSH chains leads to formation of corner states inside a band gap. Similar to the 1D case, different corner states can be stabilized by the same pump due to the nonlinearity. These results are obtained in a specific physical platform, the microcavity polariton system. However, it is important to emphasize that the topological structure and the topological states (edge states in 1D and corner states in 2D), introduced in the present work are of very general nature and can be realized in different physical implementations.

## 2 Lattices, Chern index, and theoretical model

Let us begin our discussion with the definition of the proposed DW structure and an analysis based on a generally applicable tight-binding (TB) model Hamiltonian. Further below we will then proceed to analyze a specific physical realization in the form of a planar semiconductor microcavity system and optical excitations therein.

We assume 2D DW chains with potential wells that are distributed in the *x* − *y* plane at (*x*_*n*_, ±*y*_*n*_) with *n* ∈ [1, *N*]. The positions of the potential wells are given by

(1) $$x_{n}=a\left(n-1\right),\;\;y_{n}=\frac{A}{2}+d-d\sin\left(\left(n-1\right)\frac{\pi }{2}+\theta \right).$$

Here, *a* is the separation of the neighbouring wells along the horizontal (*x*) direction, i.e., intra-wave separation, *A* represents the separation of the closest wells in the upper and lower waves, i.e., inter-wave separation, and *d* is the amplitude of each modulated wave. Figure 1(a) shows the potential distribution with the wave constant 4*a*, i.e., each four potential wells form a wave unit, and there are four periods along *x* direction with a total of 16 potential wells. The separation of the upper and lower waves, center to center, is *A* + 2*d*. The phase shift *θ* (∈ [0, 2π]) in Eq. (1) indicates the phase of the first (leftmost) well in the modulation and *θ* = 0 in Figure 1(a). In the DW chain, the profile of each individual potential well satisfies

(2) $$V\left(x,y\right)=\underset{n}{\sum }V_{0}e^{-{\left(\frac{{\left(x-x_{n}\right)}^{2}+{\left(y\pm y_{n}\right)}^{2}}{w^{2}}\right)}^{10}}.$$

Here, *V*_0_ is the potential depth and *w* radius of each potential well.

Heuristically, this DW chain can be described by a TB model with Hamiltonian

(3) $$\begin{array}{cc} \hat{H} & =\overset{N-1}{\underset{n}{\sum }}\left(J_{n,n+1}A_{n}^{†}A_{n+1}+J_{n,n+1}B_{n}^{†}B_{n+1}+h.c.\right)\\ & \;+\overset{N}{\underset{n}{\sum }}\left(t_{n,n}A_{n}^{†}B_{n}+h.c.\right) \end{array}$$

Here only the fundamental mode of each well and their nearest neighbour couplings are considered. $X_{n}^{†}\left(X_{n}\right)$ is the creation (annihilation) operator on the *n*th site (*X* = *A*, *B*) and *A*(*B*) corresponds to the upper (lower) chain. Furthermore, the Hamiltonian $\hat{H}$ has mirror exchange symmetry with the corresponding exchange operator $\hat{M}$ defined as $\hat{M}\psi =\psi^{\prime }=\left(\begin{array}{c} B_{n}\\ A_{n} \end{array}\right)$ . The Hamiltonian can be block-diagonalized onto the basis of $\hat{M}$ under the transformation $U\hat{H}U^{-1}={\hat{H}}_{BD}$ , in which ${\hat{H}}_{BD}=\left(\begin{array}{cc} h_{+} & 0\\ 0 & h_{-} \end{array}\right)$ . Finally, if we assume the inter-wave couplings to take the form *t*_*n*,*n*_ = *t* + *λ*cos (2π*βn* + *θ*) where *β* = *p*/*q* is a rational parameter (*p* and *q* are co-prime), then *h*_+(−)_ is inscribed as an AAH model, while ${\hat{H}}_{BD}$ governs two uncoupled AAH models with oppositely signed modulation. The Chern index associated with each energy band can be defined in the synthetic 2D parameter space (*θ*, *k*_*x*_) for each mirror subspace as *C*_±,*j*_ for *j* = 1, …, *N*. Here +(−) refers to the even(odd) subspace and *j* is the index of bands whose energies are arranged in ascending order. Owing to the reversely signed modulation between the two subspaces, the relation *C*_+,*j*_ = *C*_−,*N*−*j*+1_ is satisfied [see the Supplementary Materials]. Considering an individual wave unit with *N* = 4, we have *C*_+,*j*_ = *C*_−,*N*−*j*+1_ = {1, − 3, 1, 1} (the expression for the calculation of the Chern index is given in the Supplementary Materials), evidencing that the band gaps in such DW chains are topologically nontrivial.

The TB model discussed above gives general and valuable insight into the topology of states expected in the proposed DW chains, similarly applicable to any physical realization. In the following, however, we will go a step further and analyze a specific physical realization of the DW structure and resulting topological states in a semiconductor microcavity polariton system. This analysis includes the dynamical system evolution to optically excite and populate the topological states as well as the interplay of topology and nonlinearity.

To study the dynamical evolution of polariton condensates in the topologically nontrivial DW chains with resonant excitation, we consider a generalized Gross–Pitaevskii (GP) equation with loss and gain, i.e.,

(4) $$\begin{array}{cc} i\hbar \frac{\partial \psi \left(r,t\right)}{\partial t} & =\left[-\frac{\hbar^{2}}{2m}\nabla^{2}-i\hbar \frac{\gamma }{2}+g|\psi \left(r,t\right){|}^{2}+V\left(r\right)\right]\\ & \;\times \psi \left(r,t\right)+E\left(r,t\right). \end{array}$$

Here, *ψ*(**r**, *t*) is the polariton wavefunction, *m* = 10^−4^ *m*_e_ (with free electron mass *m*_e_) is the effective polariton mass, *γ* is the loss rate in quasi-mode approximation [57], and *g* is the nonlinearity coefficient representing the strength of the polariton interaction. *E*(**r**, *t*) is a coherent pump for the excitation of polaritons. *V*(**r**) represents the DW potential chain which can be fabricated in semiconductor microcavities in different ways [27]. For this physical realization, we chose *V*_0_ = −5 meV for the potential depth and *w* = 1 μm for the radius of potential wells. These parameters are based on typical experiments in GaAs-based semiconductor microcavities [27]. However, we note that the analysis and the GP model are similarly applicable to atomic condensates and nonlinear optics.

## 3 Edge states in DW chains

We start by studying the linear eigenstates of the DW chain. The eigenvalue problem can be obtained by substituting the ansatz *ψ*(**r**, *t*) = *ψ*(**r**)*e* ^−i*ωt*^ into Eq. (4) and neglecting the loss, gain, and nonlinearity. From the eigenenergies in Figure 1(b), one can see that the gapped edge states (red lines) become more isolated from the bulk states (black lines) when the intra-wave separation *a* is larger than the inter-wave separation *A*, akin to the appearance of the edge states in SSH chains when the intra-cell coupling is larger than the inter-cell coupling [58]. When the edge states blend into the bulk states (energy bands), they become topologically trivial. Note that in order to clearly distinguish the energy bands and band gaps, the spectra shown in Figure 1(b) are calculated for 13 unit cells (52 potential wells in each wave) instead of the case shown in Figure 1(a) with only 4 unit cells. However, the topological properties in both cases remain the same [cf. Figure S3(a) which is calculated with only 4 unit cells]. Since there are 8 potential wells in each unit cell, there must be 8 energy bands instead of 6 as shown in Figure 1(b). Note that two energy bands are outside the shown energy range (one with a lower energy and the other with a higher energy, cf. Figure S8.)

**Figure 2:**
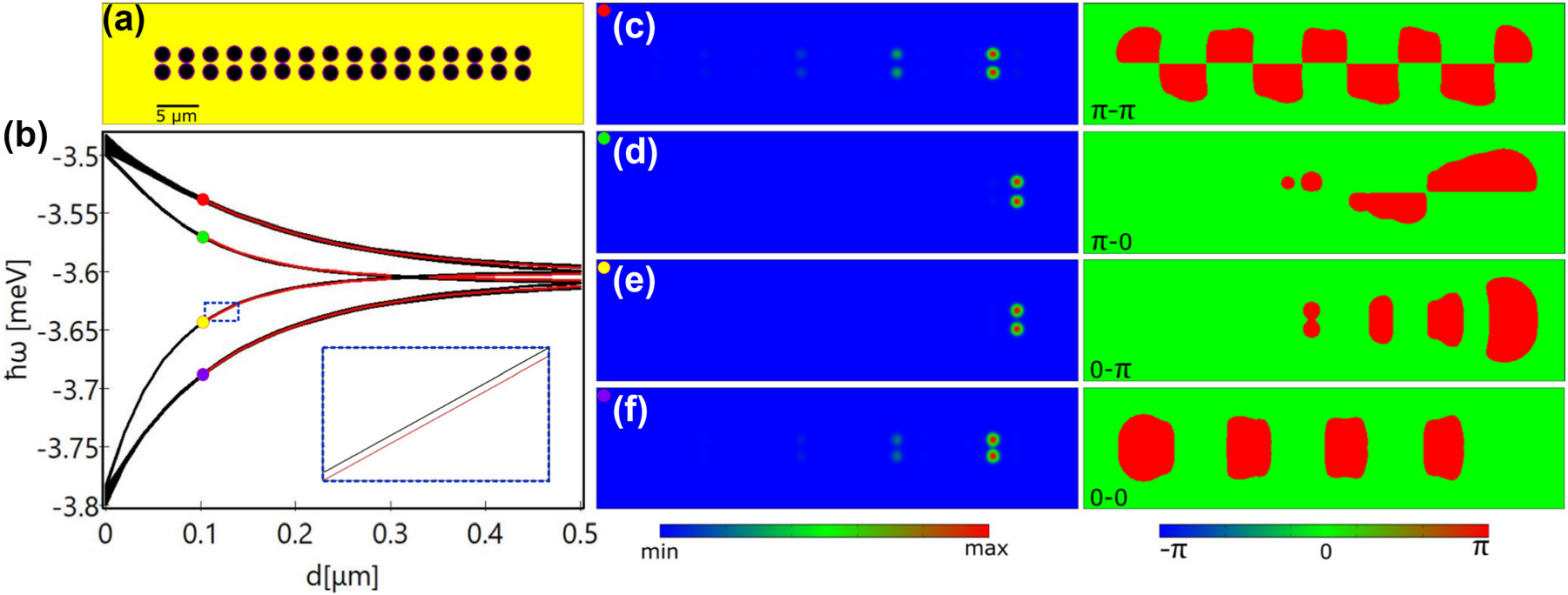
Influence of the modulation amplitude of the DW chain on the edge states. (a) Distribution of the DW potential chain with *a* = 3 μm, *A* = 2 μm, and *d* = 0.1 μm. (b) Dependence of the eigenenergies on the modulation amplitude *d*. Here *A* = 2 μm and *a* = 3 μm. Red lines indicate the topological edge states and black lines are the bulk states. (c–f) Density (left) and phase (right) distributions of the eigenstates in the DW chain presented in (a), corresponding to the symbols in (b), respectively.

In this configuration, all the edge states are located at the right edge of the chain. For each edge state four potential wells are occupied, and there are in total four different types of edge states as shown in Figure 1(c–f). These edge states can be grouped into two subgroups, i.e., 0-states [Figure 1(d and f)] and π-states [Figure 1(c and e)]. For 0-states the phases in the equivalent wells in the two waves are the same, while for π-states there is a π-phase difference between them. In each group, there are two edge states where one shows the same phase for the last two wells in the same wave [Figure 1(e and f)] and the other shows a π-phase jump [Figure 1(c and d)]. Therefore, we denote the four edge states as 0–0 state [Figure 1(f)], π-0 state [Figure 1(e)], 0-π state [Figure 1(d)], and π–π state [Figure 1(c)]. It is worth noting that in the present work both types of localized states [Figure 1(c–f)] are defined as edge states. That is, the edge states generally refer to the localized states that emerge in only the edge unit cells with the bulk unit cells empty. Besides the separation of the two waves, the properties of the edge states are also related to the modulation amplitude of the waves *d*. As *d* decreases, the inter-wave interaction in the whole chain becomes stronger, leading to edge states that are less distinguishable from the bulk states as shown in Figure 2(b). Here, the modulated waves cannot be easily recognized, that is, the two chains are almost parallel to each other [Figure 2(a)]. As a result, the 0–0 state and π–π state slightly permeate through the bulk potential wells [Figure 2(c and f)] and move deep into the bulk states [Figure 2(b)], whereas the π-0 and 0-π edge states become more localized in the edge potential wells [Figure 2(d and e)]. These two states remain in the band gaps as can be seen from the inset in Figure 2(b), although the broad frequency range of Figure 2(b) makes them difficult to be distinguished from the bulk states. Such edge states remain as more potential periods are added to the left or right side of the chain. We note that our study shows representative results for a specific choice of system parameters. Band gaps can be broadened significantly by increasing the potential depth and hopping between adjacent potential wells can be enhanced by moving them closer together [27].

**Figure 3:**
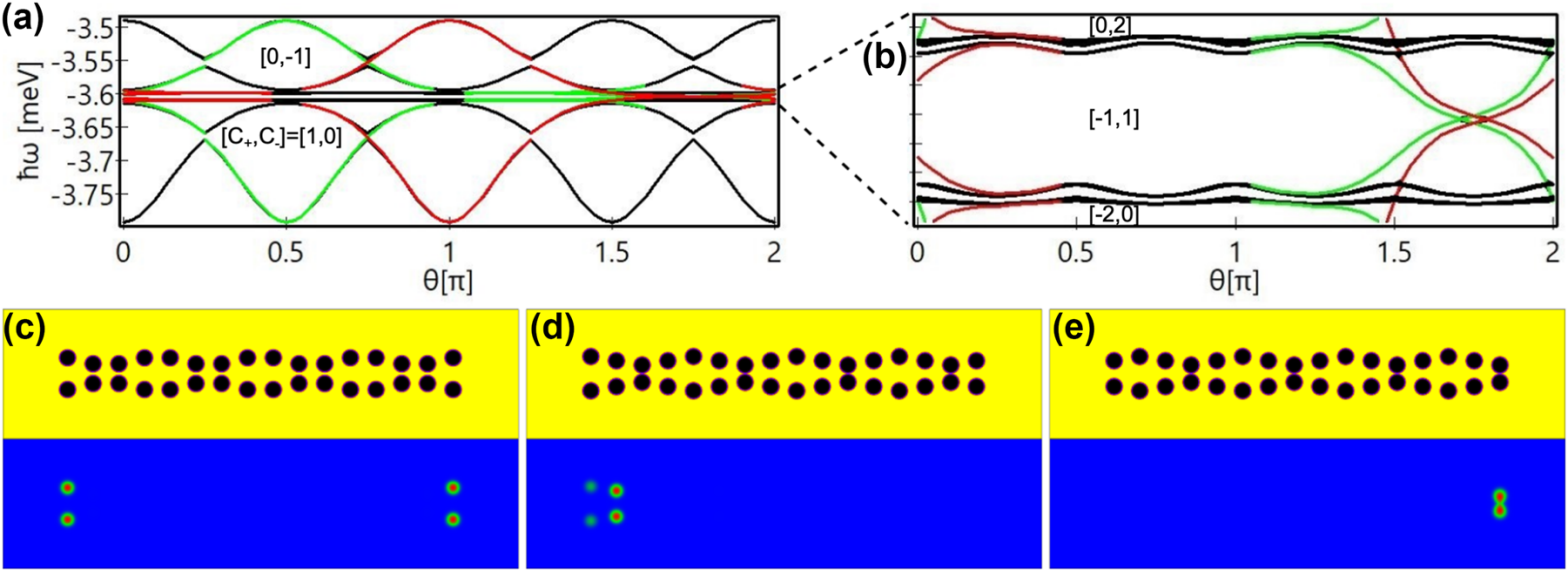
Edge states in differently structured DW chains. (a) Dependence of the energies of the linear eigenstates on the phase shift *θ* of the chains, with *a* = 3 μm, *A* = 2 μm, and *d* = 0.5 μm. Red lines indicate the right edge states, green lines indicate the left edge states, and black lines are the bulk states. (b) Enlarged view of the selected area in (a). The Chern indices have been added into the corresponding band gaps. The detailed calculation can be found in the Supplementary Materials. (c–e) Different DW chain structures (upper panels) and amplitudes [same colorbar as in Figure 1(c–f)] of the corresponding selected edge states (lower panels) at (c) *θ* = 1.75π, (d) *θ* = 1.5π, and (e) *θ* = π.

The reason why the edge states in Figures 1 and 2 appear at the right edge rather than the left one is the specific arrangement of the potential wells. How the potential wells are arranged determines which edge (left or right or both) is occupied as illustrated in Figure 3 in which the phase shift *θ* is systematically varied in panels (a) and (b) from zero to 2π. When *θ* = 1.75π, the chain becomes symmetric along *x* direction [Figure 3(c)], and consequently both edges are occupied. In this case there appear two pairs of degenerate edge states [cf. Figure 3(b)], because the wavefunctions at the two edges are spatially well separated from each other with virtually zero overlap, resulting in the degeneracy of the 0–0 and 0-π states and π-0 and π–π states. When *θ* = 1.5π [Figure 3(d)], the chain is spatially reversed in *x* direction, compared with the one in Figure 1(a). Under this condition the four edge states relocate to the left edge of the chain. When the chain is in the configuration shown in Figure 3(e) where *θ* = π, only two edge states that are localized in the rightmost wells appear. From the results presented in Figure 3 (see also the extended version of this figure, Figure S1, in the Supplementary Material), it is clear that the edge states arise only when the edge wells are not at the exact antinodes of the DW structure, i.e., only when $\sin\left[\left(n-1\right)\frac{\pi }{2}+\theta \right]\neq 0$ in Eq. (1). That is why the left edge in Figures 1 and 3(e) and the right edge in Figure 3(d) are unoccupied. The appearance of the edge states shown in Figure 3 is consistent with the inscribed Chern index calculated from the tight-binding model, Eq. (3), and thus referred to the bulk-edge correspondence [59]. The edge states can also be reconstructed by directly adding or cutting specific potential wells at the left or right side of the chain in Figure 1(a). Several examples are given in the Supplementary Material (Figure S2).

## 4 Multistable edge states

To optically excite the edge states and study their nonlinear dynamics, a resonant continuous-wave (CW) pump *E*(**r**, *t*) = *E*_0_ *e* ^−i*ωt*^, where *E*_0_ is the constant amplitude and *ω* is the frequency, is used to continuously drive the system until the stationary solution is reached. In our calculations, the polaritons have a loss rate of *γ* = 0.005 ps^−1^ and a nonlinearity coefficient of *g* = 2 μeV μm^2^. The time evolution of Eq. (1) is computed by using a 4th order Runge–Kutta method with zero boundary conditions. The stability of the stationary solutions is tested by adding white noise into the solutions and letting them evolve over 10 ns.

**Figure 4:**
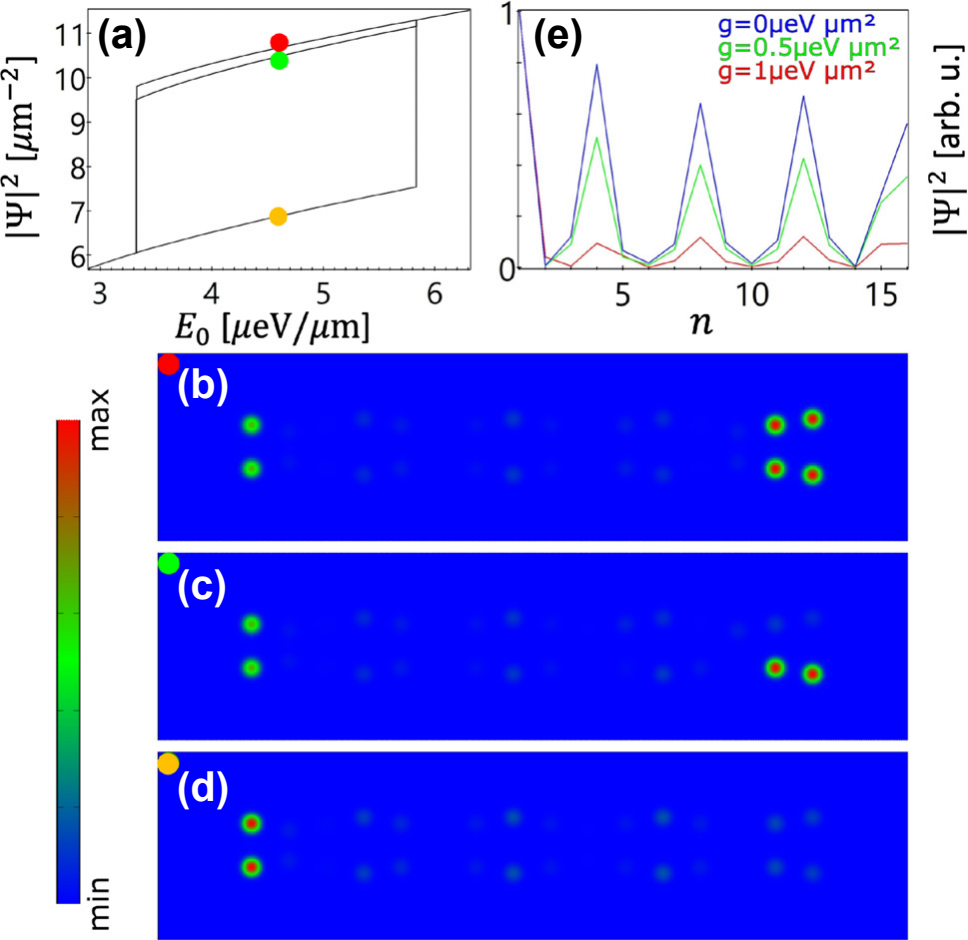
Nonlinearity and multistable edge states. (a) Dependence of the peak densities of the edge states on the amplitude of the continuous wave, spatially homogeneous pump with photon energy *ℏω* = −3.5995 meV [indicated by the red square in Figure 1(b) for *a* = 3 μm, *A* = 2.5 μm, *d* = 0.5 μm, and *θ* = 0]. (b–d) Density profiles of multistable edge states corresponding to the points marked in panel (a). (e) Normalized peak densities in each potential well from the upper wave of the state in (d) for different nonlinear coefficients *g*.

From Figure 1(b), one can see that two edge states are degenerate at around *a*/*A* = 1.17. Therefore, we choose the inter-wave separation of the chain at *a*/*A* = 1.2, where the 0-π and π–π states are very close to each other, and tune the photon energy of the pump to *ℏω* = −3.5995 meV [see the red square in Figure 1(b)]. In this scenario, because of the finite polariton lifetime and nonlinearity, the 0-π and π–π states can be simultaneously excited, and their phase difference leads to destructive interference of the condensate density in the upper wave [see the density at the right edge in Figure 4(c)]. Meanwhile, the left edge is also occupied by the condensate; however, this state is not one of the linear eigenstates as known from Figure 1. This edge state can even be separately excited as shown in Figure 4(d) especially when the pump intensity is lower. To illustrate this point further, we use the left-edge state shown in Figure 4(d) as the initial condition and gradually decrease the nonlinear coefficient while keeping the pump fixed. It is clearly seen from Figure 4(e) that the nonlinearity strongly affects this edge state. With the decrease of the nonlinearity, the condensate starts to move to the bulk of the system, forming a progressively decreasing density distribution from left to right [the blue line in Figure 4(e)], which is similar to the linear eigenstates with frequency below the 0-π edge state [see the eigenstates in the Supplementary Material Figure S3]. In conclusion the nonlinearity pushes the bulk states to the edge, leading to the formation of nonlinearity-enhanced edge states, analogous to surface gap solitons [60]. As the pump intensity increases [Figure 4(a and b)], the right edge can be fully excited, which means only one of the intrinsic edge states is excited here. If we excite the edge state in the lattice with *a*/*A* = 1.5, where the edge states are more isolated from the bulk states, by choosing the photon energy of the pump between the 0-π and π–π states, it is possible that the condensate is loaded only into the wells at the right edge, leaving the potential wells at the left edge empty [see Figure S4 in the Supplementary Material]. From Figure 4(a), one can see that all these edge states can be stable in a broad pump intensity range. We note that this finding of edge-state multistability is of general nature in our structure as it can occur at different lattice constants and pump frequencies [cf. Figure S4].

## 5 Corner states in multi-wave lattices

**Figure 5:**
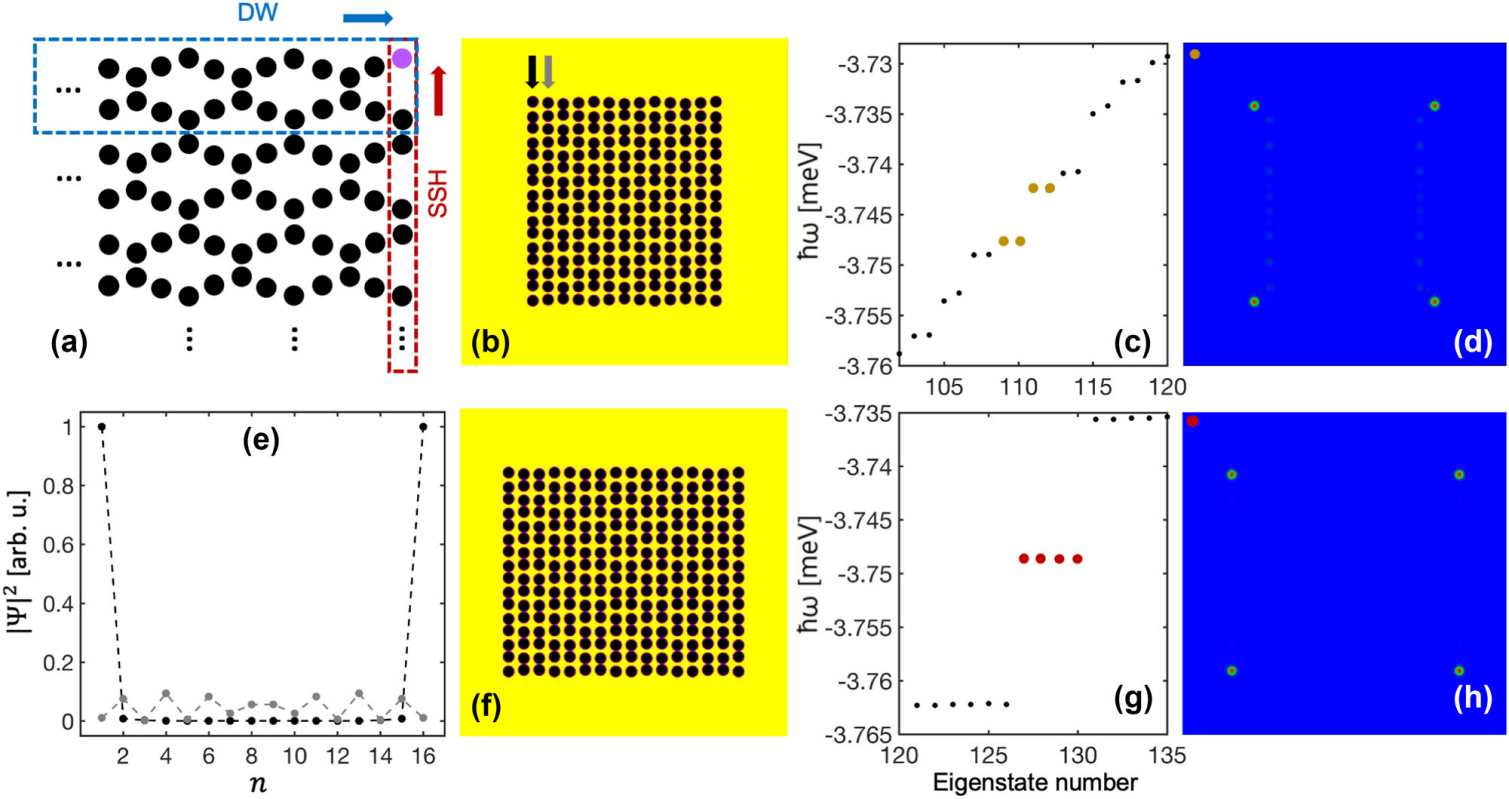
Corner states. (a) Schematic of multiple DW chains arranged along the vertical direction and the illustration of the formation of a corner state. (b) A 2D lattice pattern with *a* = 2.8 μm, *A* = 2 μm, and *d* = 0.2 μm, and the unit DW chain has the similar structure of that in Figure 2(a). (c) Selected linear eigenstates (energy vs. number) of the lattice in (b). (d) Density distributuion of the highlighted eigenstate 109 in (c). (e) Normalized peak densities (in each potential well) of the condensates, collected respectively from the two columns indicated by the two arrows in (b). (f) A 2D lattice pattern with *a* = 2.8 μm, *A* = 2 μm, and *d* = 0.2 μm, and the unit DW chain has the same structure of that in Figure 3(c). (g) Selected linear eigenstates (energy vs. number) of the lattice in (f). (h) Density distributuion of the highlighted eigenstate 127 in (g).

Although one of the corners of the DW chain in Figure 4(c) is occupied, it is not a higher-order topological insulator state, because it is induced by nonlinearity instead of a system’s eigenstate. To realize higher-order topological insulators, we extend a chain along the vertical direction, i.e., perpendicular to the chain direction, with multiple repetitions to generate a 2D lattice. Examples can be found in Figure 5(a, b, and f), in which the separation of the DW chains is always the same as the wave separation in each individual DW chain, i.e., *A* + 2*d*. From the schematic in Figure 5(a), one can see that the DW chains along the horizontal direction allow the polaritons to condense in the entire right-edge potential wells as studied above. However, the potential distribution in every column of the lattice can be regarded as an SSH chain. When the SSH chain at the right (or left) edge has a larger inter-cell coupling as illustrated in Figure 5(a), another kind of topologically nontrivial edge state is expected. As a consequence, the combination of the DW chains in the horizontal direction and the SSH chains in the vertical direction can give rise to the emergence of a corner state, that is, the polariton condensate can be solely loaded into the corner potential well marked in purple in Figure 5(a).

For the realization of such corner states, we first choose a DW chain with a smaller inter-wave separation to enable almost all the polaritons to condense in the rightmost (or leftmost) potential wells, i.e., reducing the density of the condensate in the potential wells next to the edges, referring to the edge states shown in Figure 2(d and e). The reason for reducing the condensate population in the bulk potential wells as much as possible is that in the SSH chain next to the right edge in Figure 5(a), the inter-cell coupling is prominently reduced, so that the edge states in this potential column may vanish and the condensates in the bulk potential wells would hinder the formation of a clean corner state. By extending a DW chain with a similar structure of the one shown in Figure 2(a) (but symmetric distribution along the horizontal direction) into 2D (8 repetitions along the vertical direction), a lattice containing multiple potential waves is created as shown in Figure 5(b). Figure 5(c) presents a part of the eigenstates including the corner states as highlighted. It can be seen that the band gap here is not very prominent due to the small wave modulation *d* as illustrated in Figure 2(b). What is intriguing is that the four corner states share two different energies instead of one. This is because there are still polariton condensates being loaded into the two columns adjacent to the two vertical edges, which can be vaguely seen in Figure 5(d). A clearer picture is presented in Figure 5(e) where the peak condensate densities in each potential well are extracted from the left-edge chain and the chain next to it, indicated by the two arrows in Figure 5(b). A small amount of condensate appears in the bulk potential wells, and the energy difference of the four corner states originates from the phase difference (0 or π, see Figure S5 in the Supplementary Material) of the condensates in these two adjacent chains.

To completely remove the influence of the weak polariton density in the bulk potential wells on the corner states, we focus on the DW distribution shown in Figure 4(c) in which only two edges are occupied. Extending this chain along the vertical direction gives a 2D lattice pattern shown in Figure 5(f). In this case, because of the vanishing of the polaritons in the bulk potential wells, the condensates in the four corner potential wells becomes completely disconnected [Figure 5(h)], so that the four edge states are degenerate in energy [Figure 5(g)]. The band gap here is broader than that in Figure 5(c) because of the increased wave modulation *d*. Apart from the 0D corner states, there exist also 1D edge states in such 2D lattices, examples can be found in Figure S6 in which the dependence of the topological states and the wave modulation *d* is also illustrated. It is known that when the intra-cell coupling is stronger than the inter-cell coupling in an SSH chain, the topological edge states disappear. Therefore, if the top single-wave chain in Figure 5(a) is removed, the corner state will be erased accordingly from the system. An example is given in the Supplementary Material Figure S5, in which the lattice is obtained by extending the DW chain in Figure 3(e) into 2D. In this scenario, a SSH chain with a larger intra-cell coupling is formed at the right edge, hence the corresponding band gap is empty.

**Figure 6:**
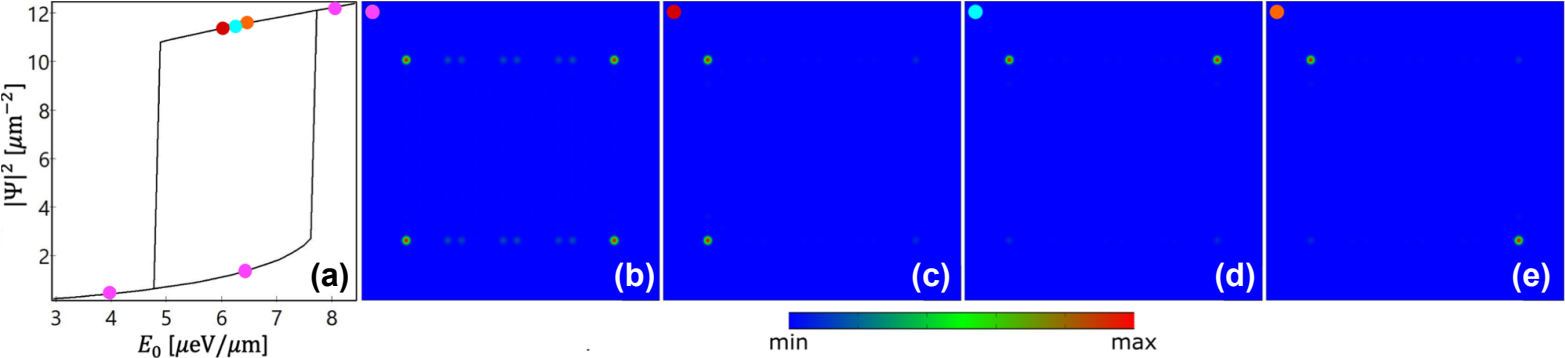
Multistable corner states. (a) Dependence of the peak densities of the corner states in the lattice presented in Figure 5(f) on the amplitude of the continuous wave, spatially homogeneous pump with photon energy *ℏω* = −3.7449 meV. (b–e) Density profiles of multistable corner states corresponding to the points marked in (a).

The corner state presented in Figure 5(h) can also be optically excited by a homogeneous pump with a near resonant photon energy. Similar to the case studied in the 1D DW chain in Figure 4, the nonlinearity can support more than one stable corner state as illustrated in Figure 6. That is, besides the intrinsic individual eigenstate with four corners [Figure 6(b)], the combination of any two different eigen corner states can also be excited due to the nonlinearity, resulting in new corner states with only two corners being excited as can be seen in Figure 6(c–e). Due to the missing two corners, these states have a higher shared density than that of the four-corner state under the same pump in the multistability region [see Figure 6(a)]. Outside this region, only the four-corner states exist.

## 6 Conclusion and discussion

In summary, we report on different topological edge states in polaritonic DW chains. Their topological nature is evidenced by the nonzero Chern index in the linear regime. Under specific pumping conditions more than one edge state can be stabilized, including the combination of different intrinsic edge states as well as nonlinearity-enhanced ones. These states can appear at different edges for differently structured DW potentials. We also demonstrate that SSH chains can occur in multiple coupled DW chains (2D lattices), ascribed to the spatial wave distribution of the potential wells, and their direction is perpendicular to that of the DW chains, which support 0D localization in a band gap. Multistable corner states are also studied in the nonlinear regime. To demonstrate the robustness of the edge and corner states, we include a strong defect, by completely removing a corner potential well, in both 1D and 2D cases to study its influence on these interesting topological states and find that the edge and corner states are very robust and show stable time evolution in the presence of a defect, without evolving into bulk states (see Figure S7 in the Supplementary Material).

Although implemented in a specific system, our theoretical model and main results are of general nature. Similar observations of topologically protected edge and corner states in these structures are expected in other physical realizations such as solid-state and photonic systems utilizing the advantages of our structure. For example, the 1D DW chains, comparing with the SSH and AAH chains, enable the emergence of different edge states and their combinations can be stabilized by nonlinearity, giving rise to multistable edge states. The switching between the multistable edge states for example, which is beyond the scope of this work, could benefit the development of all-optical switches. In polariton condensates, the topological edge states in the SSH chains occur at higher-order modes (dipoles) with higher energy, instead of fundamental modes [8], [35], [36], while in the DW chains the topological edge states appear in the fundamental modes with lower energy.

## Supplementary Material

Supplementary Material Details
