# Supplementary material for: Topological edge and corner states in coupled wave lattices in nonlinear polariton condensates
Source: Nanophotonics. 2024 Feb 2;13(4):509–18. doi: 10.1515/nanoph-2023-0556 (PMC11501762; doi:10.1515/nanoph-2023-0556)
Supplement: Supplementary file 1 — Supplementary Material Details [file j_nanoph-2023-0556_suppl_001.pdf]

Supplementary Material:

Topological edge and corner states in coupled wave lattices in nonlinear polariton condensates

Tobias Schneider,[1] Wenlong Gao,[2] Thomas Zentgraf,[1,3] Stefan Schumacher,[1,3,4] Xuekai Ma,[1]

[1]Department of Physics and Center for Optoelectronics and Photonics Paderborn (CeOPP), Paderborn University, Warburger Strasse 100, 33098 Paderborn, Germany

[2]EIT Institute for Advanced Study, Ningbo, China

[3]Institute for Photonic Quantum Systems (PhoQS), Paderborn University, 33098 Paderborn, Germany

[4]Wyant College of Optical Sciences, University of Arizona, Tucson, AZ 85721, USA

I. Configuring different edge states

Figure S1 is the extended version of Fig. 2, where more examples of the chain distributions and the corresponding edge states are shown. It is clearly seen that the edge states appear as long as the edge potential wells are the most or lest separated ones in the double waves, even if the phase shift θ is an arbitary value [see Fig. S1(j)].

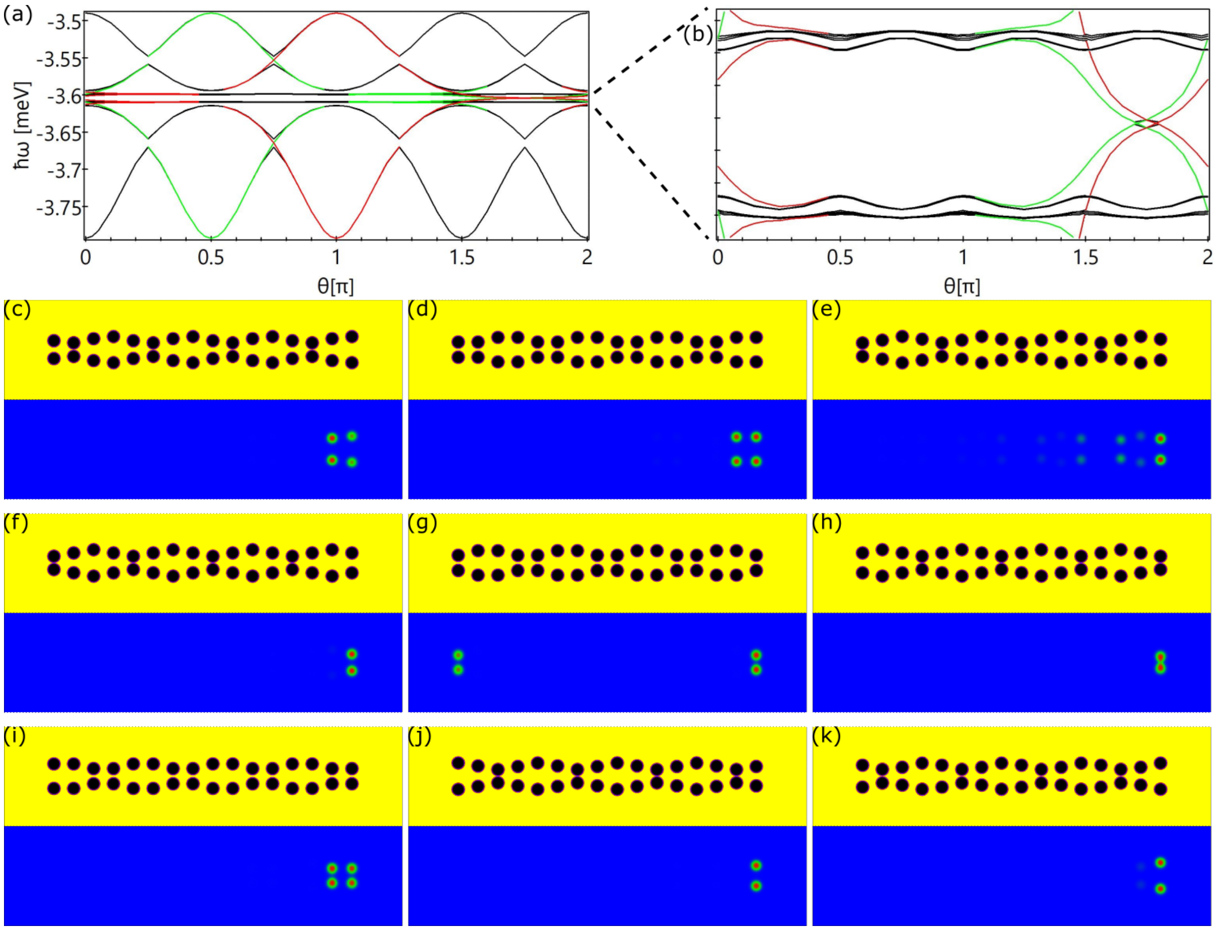

Figure S1: **Edge states in differently structured double-wave chains.** (a) Dependence of the eigenenergies of the linear modes on the phase shift of the chains θ. Here $a = 3$ μm, $A = 2$ μm, and $d = 0.5$ μm. Red lines indicate the right edge states, green lines indicate the left edge states, and black lines are the bulk states. (b) Enlarged view of the selected area in (a). (c-k) Profiles of the chain (upper) and the corresponding density of the selected edge states (lower) at different phase shifts: (c) $\theta = 0.1\pi$, (d) $\theta = 0.25\pi$, (e) $\theta = 0.45\pi$, (f) $\theta = 0.5\pi$, (g) $\theta = 0.75\pi$, (h) $\theta = \pi$, (i) $\theta = 1.25\pi$, (j) $\theta = 1.525\pi$, and (k) $\theta = 1.9\pi$.

II. Edge states in double-wave chains with different lengths

To realize different edge states, one can also cut or add some potential wells in the chain. Here we consider the chain in Fig. 1(a) as the standard one. By cutting four potential wells at the right edge as shown in Fig. S2(a), similar edge states like the one shown in Fig. S1(h) form [see Fig. S2(d,g)]. If two potential wells at the left edge is removed [Fig. S2(b)], two types of edge states appear in the same chain with one at the left edge, where the potential wells are the least separated, and the other one at the right edge, where the potential wells are the most separated [Fig. S2(e,h)]. With adding two more potential wells to the left side of the chain to make it symmetric along x direction [Fig. S2(c)], both edges are allowed to be occupied by the condensates with the same profile as shown in [Fig. S2(f,i)].

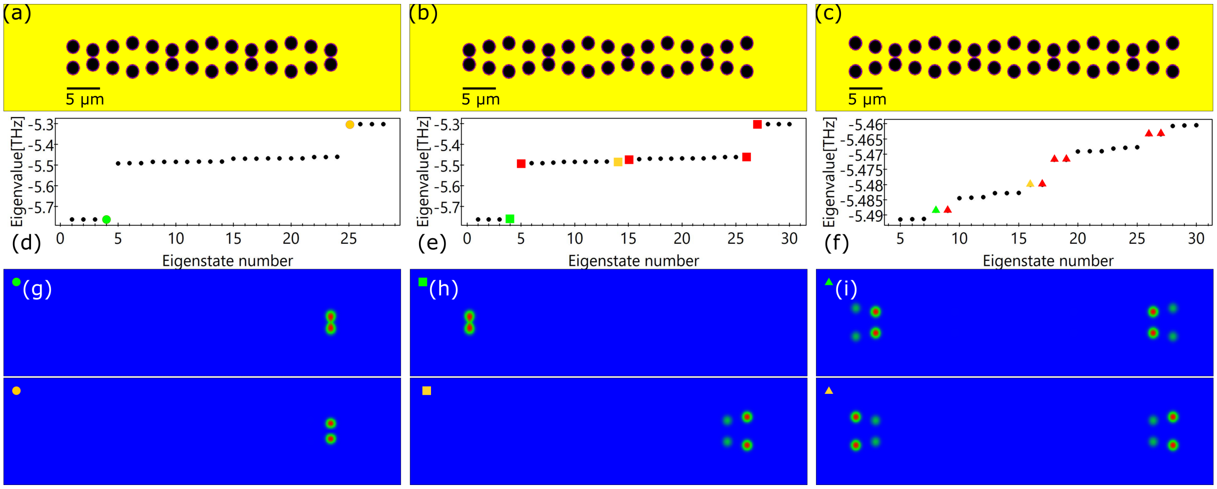

Figure S2: **Potential chains with different lengths and the corresponding edge states.** (a-c) Profiles of the double-wave chains with the same lattice parameters, $a = 3\ \mu$m, $A = 2\ \mu$m, and $d = 0.5\ \mu$m, but different lengths along the horizontal (x) direction. (d-f) Eigenfrequencies of the linear eigenstates versus the eigenstate numbers. (g-i) Selected edge states of the chains (a-c), corresponding to the symbols in (d-f), respectively.

III. Eigenstates at the inter-wave separation $A = 2.5$ μm

When the inter-wave separation $A = 2.5$ μm, the density of the condensate of the edge states in the four potential wells at the right edge are almost identical [Fig. S3(b,c)]. Figure S3(d-g) shows the eigenstates (bulk states) with the energies below the two edge states. The condensates in such bulk states can be suppressed and consequently the condensate at the left edge is enhanced, forming new edge states, i.e., nonlinearity-induced edge states (see Fig. 4 in the main text).

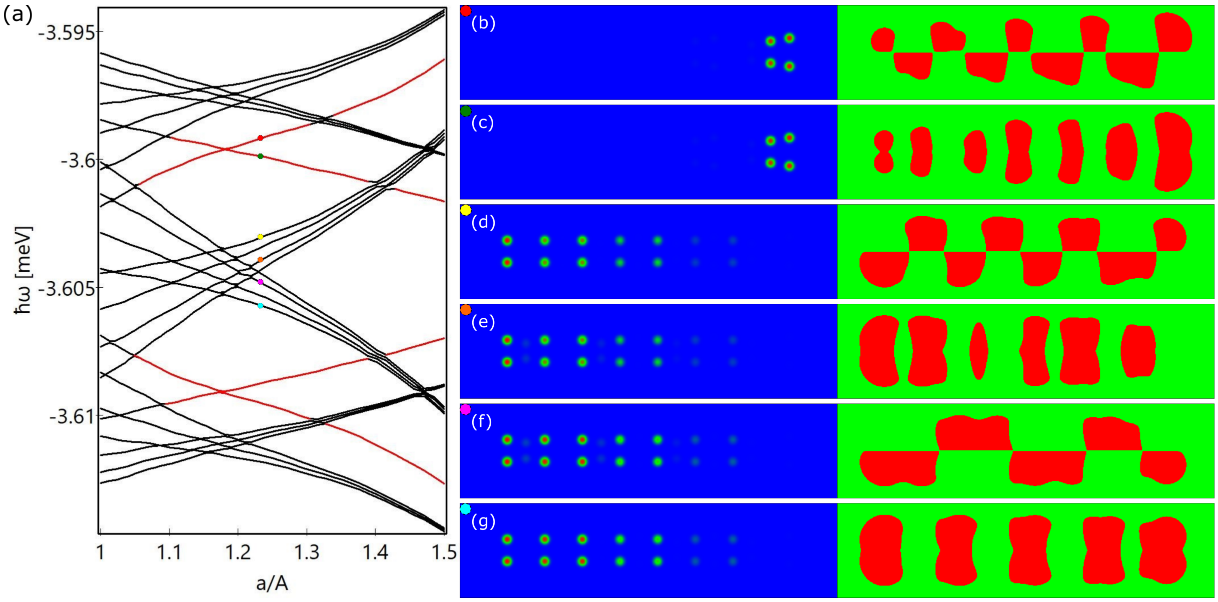

Figure S3: **Linear eigenstates.** (a) Dependence of the eigenenergies of the linear eigenstates in the double-wave chain on the inter-wave separation A, calculated based on the chain in Fig. 1(a) with $a = 3$ μm, $d = 0.5$ μm, and $\theta = 0$. Red lines indicate the edge states and black lines are the bulk states. (b-g) Density (left) and phase (right) distributions of the selected eigenstates, corresponding to the symbols in (a), respectively.

IV. Multistable edge states

From Fig. 1(b) or Fig. S3(a), one can see that when $a/A = 1.5$, the edge states are more isolated from the bulk states, so that in this case, the edge state [Fig. S4(c,f)] can be solely excited without the excitation of the left edge state [c.f. Fig. 4(b)]. The left edge can still be excited when the pump intensity is stronger as shown in Fig. S4(a,e). When the pump intensity is stronger, the 0-π edge state at the right edge is still slightly influenced by the π-π state [see the density distribution in Fig. S4(d)], even there is a significant energy gap between them. It is clear that under some specific pump intensity range, the bistability remains in this case as shown in Fig. S4(a).

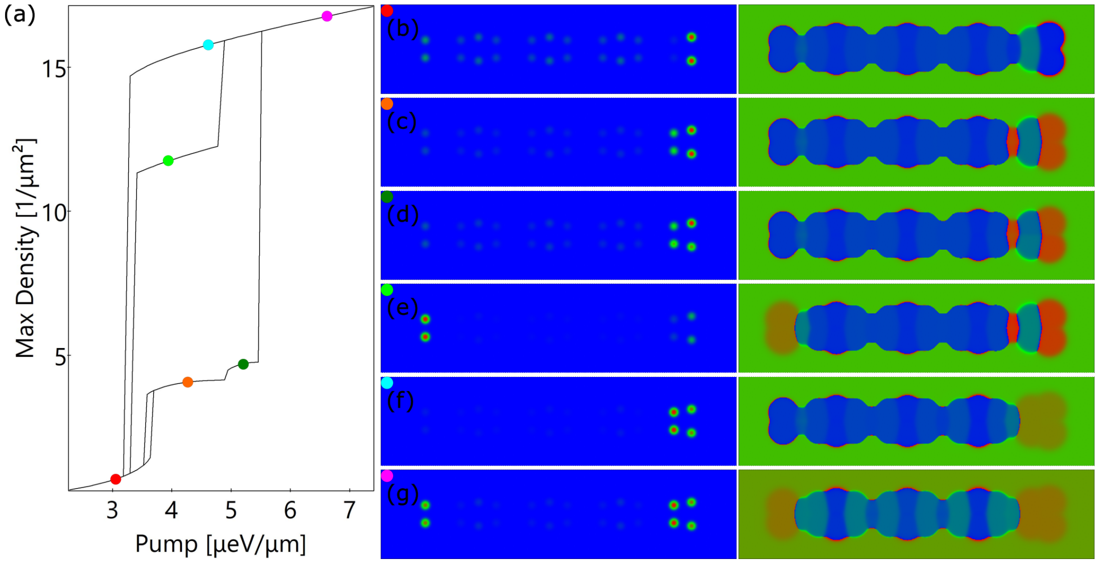

Figure S4: **Bistable edge states.** (a) Dependence of the peak density of the nonlinear edge states on the amplitudes of the pump with photon energy $\hbar\omega$ =-3.5982 meV [the chain parameters are $a = 3$ μm, $A = 2$ μm, $d = 0.5$ μm, and $\theta = 0$, the same with the one shown in Fig. 1(a)]. (b-g) Density (left) and phase (right) profiles of the states marked in (a), respectively.

V. Corner states

It can be seen from Fig. S5(a) that the four corner states share two different energies. This is because there is a very small number of condensate being loaded into the chains next to the left and right edges [Fig. S5(b)]. The coupling of the condensates in the edge potentials and the potentials next to them induces the energy difference. The lower energy represents that the condensates in the adjacent vertical chains have the same phase [see Fig. S5(c,d)], while the higher energy indicates that their phase difference is π, see Fig. S5(e,f).

If a 2D lattice has the structure shown in Fig. S5(g), in the two SSH chains at the left and right edges, the intra-cell coupling is apparently stronger than the inter-cell coupling. Consequently, the left and right edges prevent the formation of the edge states along the vertical direction, so that there is no corner states in the corresponding band gap as can be seen in Fig. S5(h).

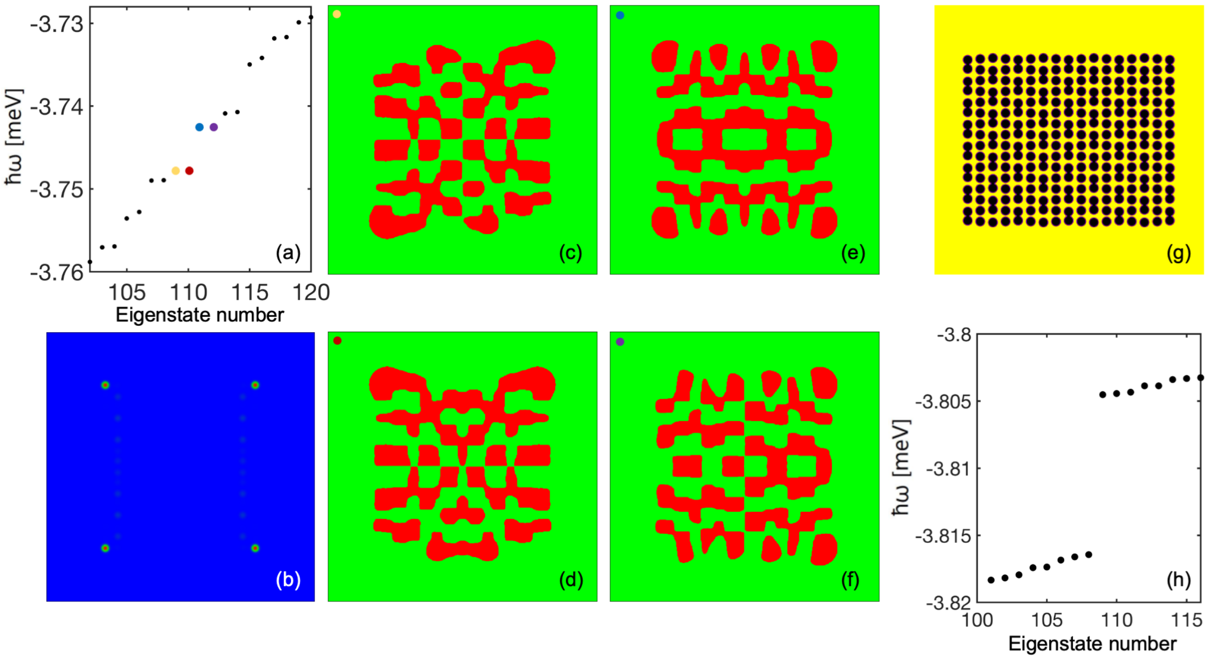

Figure S5: **Corner states.** (a) Selected linear eigenstates (energy vs. number) of the lattice in Fig. 5(b). (b) Density distribution of the corner state 109 in (a). (c-f) Phase profiles of the four corner states highlighted in (a). (g) A 2D multi-wave lattice pattern with $a = 2.8$ μm, $A = 2$ μm, and $d = 0.2$ μm. (h) Selected linear eigenstates (energy vs. number) of the lattice in (g).

In the 2D lattice presented in Fig. 5(f), there exist also 1D edge states as illustrated in Fig. S6. The 1D edge states at left and right edges [Fig. S6(b,c)] reside in the energy bands, inseparable from the bulk states, while the edge states appeared at upper and lower edges [Fig. S6(d,e)] are gapped. From the modulation amplitude d dependent spectrum [Fig. S6(f)] one can see the transition of the edge and corner states from topologically trivial to nontrivial as d increases.

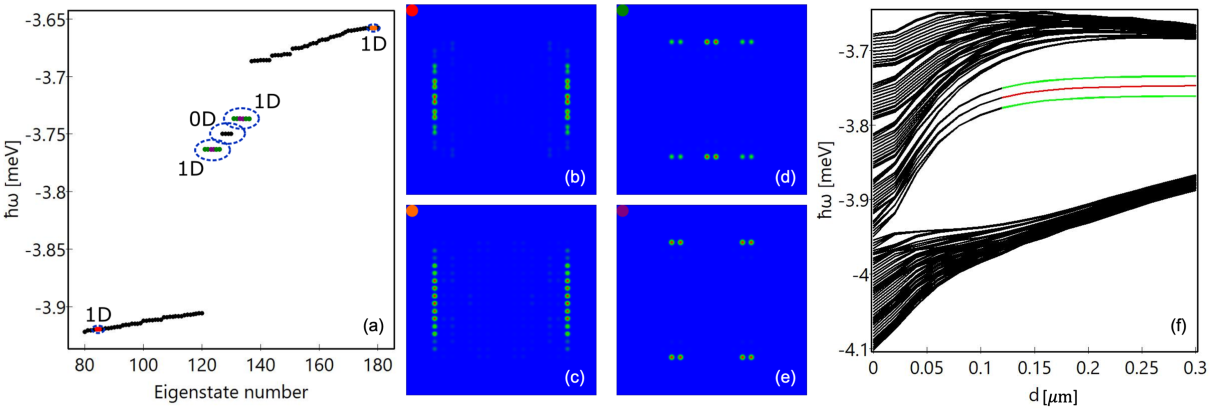

Figure S6: **Edge states in the 2D lattice.** (a) Selected linear eigenstates (energy vs. number) of the 2D lattice in Fig. 5(f). Here, 0D corner states and 1D edge states are marked. (b-e) Density distributuions of the 1D edge states highlighted in (a). (f) Dependence of the eigenenergies on the modulation amplitude d in the 2D lattice with $A = 2\mu$m and $a = 2.8\mu$m fixed. Red lines indicate the 0D corner states, green lines indicate the 1D edge states, and black lines indicate the bulk states.

VI. Robustness of edge and corner states

To demonstrate the robustness of the topological edge and corner states in the nonlinear regime, potential defects are applied to the original lattices. Figure S7(a) shows the 1D DW chain studied in the main text, but a potential well is removed from the lower wave chain. When we use the same excitation manner introduced in Fig. 4, one can see from Fig. S7(b) that the edge state, corresponding to the one shown in Fig. 4(b), can still be excited and is stable. This holds also for the 2D case as can be seen from Fig. S7(c,d) which correspond to the excitation studied in Fig. 6. These results are stable states obtained from the time evolution and evidence that the topological states studied in our work are very robust against strong defects. These topological states are also robust against significant white noise. An example is shown in Fig. S7(e,f), starting from an initial condition that despite the addition of white noise leads to stable time evolution into the state in Fig. S7(f).

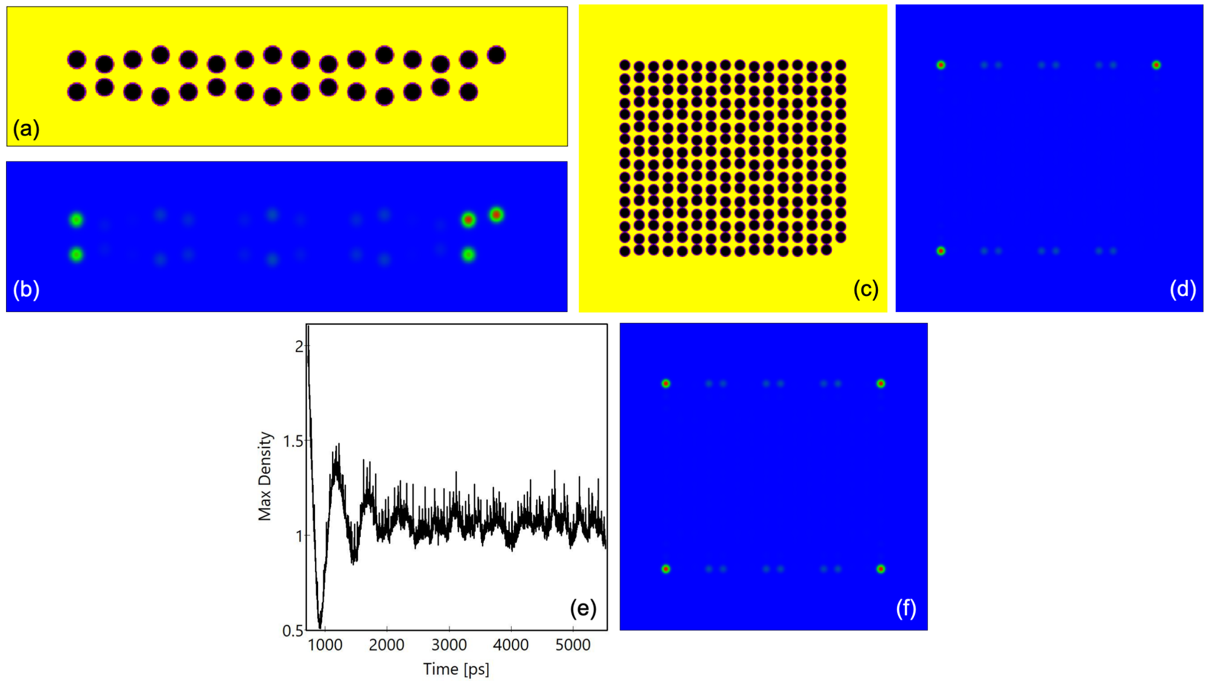

Figure S7: **Edge and corner states with potential defects.** (a) Profile of a DW chain with the same parameters of the one shown in Fig. 1(a) but a missing potential well at the right edge of the lower chain. (b) The corresponding density profile created by the same pump used in Fig. 4. (c) Profile of a 2D structure with the same parameters of the one shown in Fig. 5(f) but a missing potential well at the right bottom corner. (d) The corresponding density profile created by the same pump used in Fig. 6. (e) Time evolution of the peak density of the corner state shown in Fig. 6(b) with random white noise being added during the time evolution. (f) Density profile of the corner state at $t = 5000$ ps.

VII. Chern index of the even and odd subspaces

In this section, the Chern index relation between the even and odd subspaces is derived. Following the formula used in the main text, the vertical couplings are $t + \lambda\cos(2\pi p/(N+1)j + \theta)$ and the horizontal couplings are 1 . We know that the total Hamiltonian of two uncoupled AAH models reads: $\hat{H}_{BD} = \begin{pmatrix} h_+ & 0 \\ 0 & h_- \end{pmatrix}$. It is beneficial to write the position informed momentum space Hamiltonian of the odd subspace:

$$h_-(k_x) = t + \begin{bmatrix} \Lambda_1 & e^{ik_x/N} & 0 & \dots & 0 & e^{-ik_x/N} \\ e^{-ik_x/N} & \Lambda_2 & & & & \\ 0 & & & & \ddots & \\ \vdots & & & \ddots & \ddots & e^{ik_x/N} \\ 0 & & & & & \\ e^{ik_x/N} & 0 & \dots & 0 & e^{-ik_x/N} & \Lambda_N \end{bmatrix} . \tag{1}$$

In this equation, we have $\Lambda_j = \lambda\cos(2\pi p/(N+1)j + \theta)$. Furthermore for the even subspace we have, $h_+ : h_-|\Lambda_j \to -\Lambda_j, t \to -t$, meaning the modulations are π in phase between two subspaces. Next, eigenstates of the odd subspace satisfy $h_-|\Psi_{-,j}\rangle = E_{-,j}|\Psi_{-,j}\rangle$. It is apparent we have the following equivalence relation: $h_-(k_x)|\Psi_{-,j}(k_x)\rangle = -h_+(k_x + N\pi)|\Psi_{-,j}(k_x)\rangle = E_{-,j}(k_x)|\Psi_{-,j}(k_x)\rangle$, which leads us to equivalence:

$$\begin{aligned} E_{+,j}(k_x) &= -E_{-,N-j+1}(k_x + N\pi)\,, \\ |\Psi_{+,j}(k_x)\rangle &= |\Psi_{-,N-j+1}(k_x + N\pi)\rangle\,. \end{aligned} \tag{2}$$

This relation allows us to conclude $C_{+,j} = C_{-,N-j+1}$.

The Chern index can be calculated separately for the h_+ and h_- sectors since they are decoupled by mirror symmetry. The Chern index for the j-th band is expressed as

$$C_{\pm,j} = \frac{1}{2\pi}\int_{-\pi}^{\pi} dk_x \int_0^{2\pi} d\theta\, \mathrm{Im}(\langle\partial_{k_x}\Psi_{\pm,j}|\partial_\theta\Psi_{\pm,j}\rangle - \langle\partial_\theta\Psi_{\pm,j}|\partial_{k_x}\Psi_{\pm,j}\rangle). \tag{3}$$

In practice, we numerically evaluated the Chern index for each mirror-symmetric sector, in the tight-binding approximation, according to the well established numerical method [T. Fukui *et al.*, Journal of the Physical Society of Japan, 74, 1674-1677 (2005).]. The result is consistent with the appearance of edge states, hence the bulk-edge correpondance [Y. Hatsugai *et al.*, Physical Review Letters, 71, 3697 (1993).]. The Chern index is the preferred topological index, since in the 2D parameter space (k_x, θ), the time-reversal symmetry links (k_x, θ) to $(-k_x, -\theta)$. Despite the actual physical structure is time-reversal invariant under fixed θ. This allows us to define the 2D Chern index for such AAH-like models. Previous studies have shown that the AAH model allows the observation of topological pumping, which is determined by the Chern index [Y. Ke *et al.*, Laser & Photonics Reviews, 10, 995-1001 (2016).].

VIII. Bulk-edge correspondence

The Chern numbers of each band gap can be calculated through the bulk-edge correspondence as illustrated in Fig. S8. For each energy band, there is a Chern number which has been calculated and given in the main text, and they have been labeled to the corresponding energy bands in Fig. S8. The Chern numbers of a band gap can be calculated by accumulating the corresponding numbers of all the energy bands below it. For example, the top gap's Chern numbers [0, -1] is obtained by applying $C_+ = 1 + 1 - 3 + 1$ and $C_- = -3 + 1 + 1$. Note that the edge states, denoted by solid red lines, seems absent in the top gap, despite the nontrivial Chern index. This is due to the fact that these edge states appear at $\theta \approx 0.75\pi$ (Fig.S1), while Fig. S8 is calculated with $\theta = 0$.

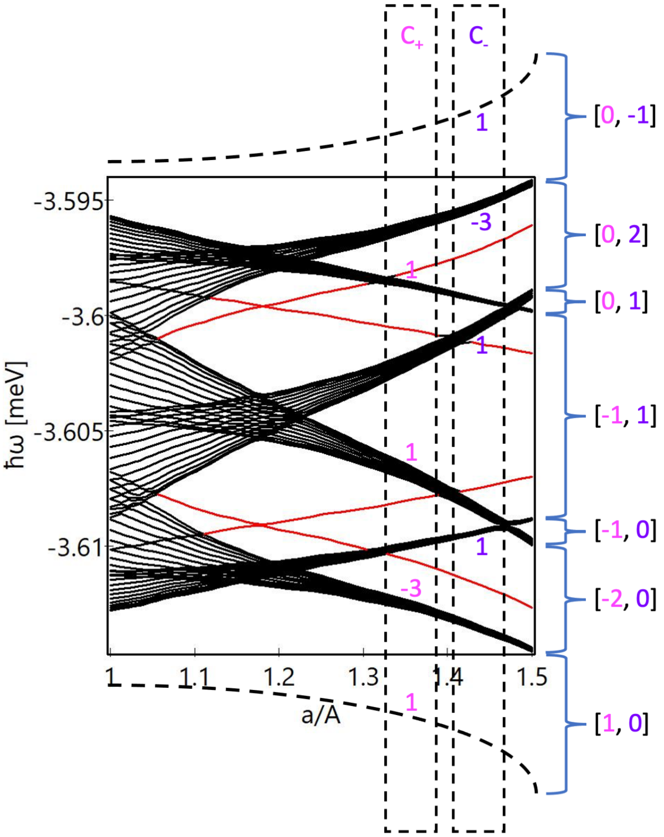

Figure S8: **Bulk-edge correspondence and the corresponding Chern numbers of the band gaps.** The energy bands are the same with that in Fig. 1(b), while the two dashed lines below and above represent the other two energy bands at much smaller and larger energies, respectively.
